# Characteristics of the parent‐child relationship at 18‐months of age predict the onset and type of mental disorders in childhood and adolescence: A longitudinal nested birth cohort study

**DOI:** 10.1002/jcv2.12304

**Published:** 2025-02-04

**Authors:** Kristine Kahr Nilsson, Lasse Grønnebæk, Ole Karkov Østergård, Susanne Landorph, Tine Houmann, Else Marie Olsen, Anne Mette Skovgaard

**Affiliations:** ^1^ Department of Communication and Psychology Aalborg University Aalborg Denmark; ^2^ Child and Adolescent Mental Health Center Mental Health Services Copenhagen Denmark; ^3^ Department of Epidemiology University of Copenhagen Section of Social Medicine Copenhagen Denmark; ^4^ National Institute of Public Health Faculty of Health Sciences University of Southern Denmark Odense Denmark

**Keywords:** early childhood, mental disorders, neurodevelopmental disorders, parent‐child relationship, parenting

## Abstract

**Background:**

The quality of the parent‐child relationship in the first years of childhood has been associated with various long‐term mental health outcomes. However, it is unclear whether specific characteristics of the early parent‐child relationship predict the onset of mental disorders. The aim of this study was therefore to examine child, parent, and dyadic characteristics of the early parent‐child relationship as predictors of mental disorders in childhood and adolescence.

**Method:**

As a part of the Copenhagen Child Cohort 2000, a child cohort of 295 children participated in the study at 18‐months of age and were subsequently followed until late adolescence. Child, parent, and dyadic dimensions of the early parent‐child relationship were assessed using the observational Parent‐Child Early Relational Assessment. The outcomes were mental disorders subsequently diagnosed in childhood and adolescence.

**Results:**

Cox‐regression analyses suggested that parent characteristics involving negative affect and behavior, intrusiveness and insensitivity during early parent child interactions were associated with increased risk of the child being diagnosed with a mental disorder at later ages. However, variation was noted across specific diagnoses. Parental negative affect and behavior was specifically associated with increased risk of attention deficit hyperactivity disorder, while infant dysregulation and irritability was specifically associated with increased risk of autism spectrum disorders. These results remained significant after adjusting for parental mental disorders and early mental health problems of the child.

**Conclusion:**

Disorder‐specific associations were observed between dysfunctions in the early parent‐child relationship and subsequent mental disorders. Improving such early relational dysfunctions may support the goals of early interventions that aim to mitigate the risk of mental disorders from a very early age.

## Graphical abstract

This study found that the characteristics of the parent‐child relationship at 18‐months of age predicted the onset and type of mental disorders in childhood and adolescence. ADHD was specifically predicted by parental negative affect and behavior during early parent‐child interactions, while autism spectrum disorders were predicted specifically by infant dysregulation and irritability.

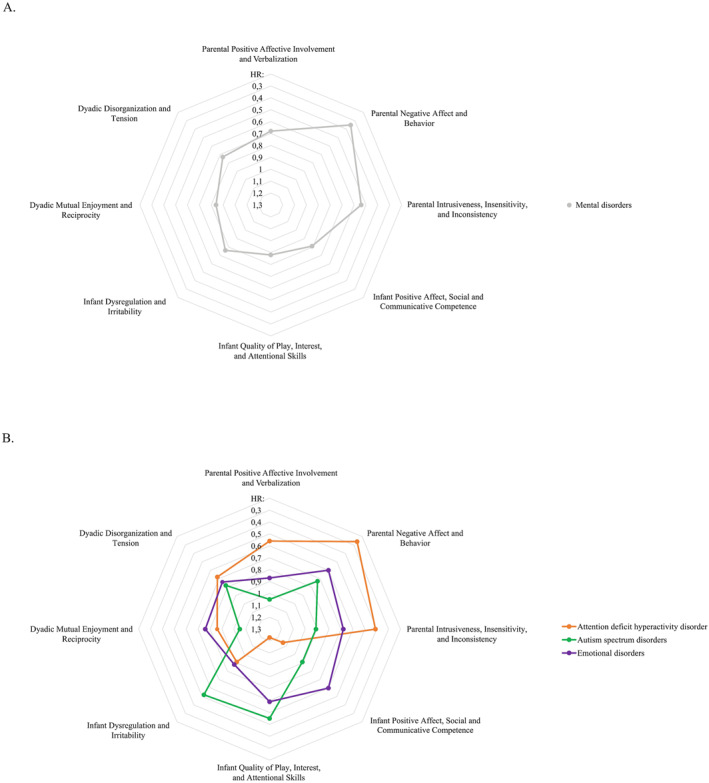

**Key points**

- While the quality of the early parent‐child relationship has been found to predict mental health outcomes at later ages, there is limited research on its predictive value with mental disorders as outcomes.
- The aim of this longitudinal study was therefore to examine whether and to what extent specific characteristics of the early parent‐child relationship, as measured at 18 months of age, predicted the subsequent onset of mental disorders in childhood and adolescence.
- Relational dysfunctions at the parental level, but not at the child or dyadic levels, predicted children's likelihood of being diagnosed with a mental disorder at later ages.
- At the same time, notable disorder‐specificity was observed, as attention deficit hyperactivity disorder (ADHD) was specifically predicted by parental negative affect and behavior, while autism spectrum disorder (ASD) was specifically predicted by infant dysregulation and irritability.
- Improving such early relational dysfunctions may facilitate early interventions that aim to mitigate the risk of these mental disorders.

## INTRODUCTION

The first years of childhood mark a period of heightened neurodevelopmental plasticity, during which children are particularly susceptible to environmental inputs, especially responses from their primary caregivers (Gee & Cohodes, 2021; Guyer et al., 2018). Through early interactions with caregivers, children acquire rudimentary forms of learning that scaffold subsequent cognitive and emotional development (e.g., Fonagy et al., 2014; McLaughlin et al., 2017). The quality of the early parent‐child relationship is therefore assumed to influence various aspects of long‐term development and mental health.

As parents and children continually influence each other during interactions, their relationship is inherently bidirectional (Sameroff & Mackenzie, 2003). At the same time, parents and children also possess individual characteristics that manifest in their interactions, shaping the dynamics of their relationships. Consequently, the parent‐child relationship may be analyzed at different levels, corresponding to the parent, the child, and their dyadic interplay (Clark et al., 2019). Each of these levels has served as a point of departure in longitudinal studies on children's mental health outcomes. Studies focusing on early relational characteristics of the child have, for instance, examined attachment styles, indicating that infants with insecure and disorganized attachment are more likely to exhibit mental health problems at later ages (Bosquet & Egeland, 2006; Carlson, 1998; Lyons‐Ruth et al., 1993). In parallel, other longitudinal studies have examined early relational characteristics of the parent, indicating that infants who experience lower parental sensitivity and responsiveness are more likely to develop internalizing and externalizing symptoms (Jacobvitz et al., 2022; Kok et al., 2013). Rather than focusing on the early relational characteristics of the child or the parent, a third group of longitudinal studies has centered on features of the early parent‐child dyad. These studies suggest that parent‐infant dyads with poorer interaction quality and lower pleasure are associated with children having more mental health problems at later ages (Dubois‐Comtois et al., 2013; Mäntymaa et al., 2015). Taken together, research on each of these relational levels adds to the understanding of early relational patterns potentially implicated in children's mental health outcomes. Still, certain questions and challenges remain to be addressed. First, most of this research has focused on either child, parent, or dyadic predictors. Considering that the parent‐child relationship is essentially dyadic but at the same time also reflects individual characteristics of the parent and the child, it is contentious as to whether any of these levels can be understood in isolation. Accordingly, a multilevel perspective that considers each of these levels in tandem provides a more complete description of the early parent‐child relationship. Second, in most of the cited longitudinal studies, mental health outcomes were measured using parent‐rated scales. Although parents tend to have valuable insights into the mental health of their child, their ratings do not translate directly to psychiatric outcomes, such as mental disorders diagnosed by qualified clinicians synthesizing various relevant information. Mental disorders are estimated to affect one in eight children and adolescents (Barican et al., 2022; Polanczyk et al., 2015; Vasileva et al., 2021) and account for a large proportion of disabilities in this age group (Castelpietra et al., 2022). Therefore, it is pertinent to identify early indicators that can inform preventive interventions. Among longitudinal studies on the parent‐child relationship in the first childhood years, only a few have focused on mental disorders as outcomes. These studies can broadly be divided into two groups, focusing either on low‐risk or high‐risk child populations. A review of the studies involving low‐risk child populations concluded that certain parent characteristics during early parent‐child interactions, such as less joint attention, as well as lower parental activity and speech, were associated with children meeting the criteria for probable mental disorders at later ages (McAndie et al., 2022). The findings summarized in this review were predominantly derived from a specific child cohort study in which parents' and teachers' ratings on the Development and Wellbeing Assessment (DAWBA; Goodman et al., 2000) were used to quantify mental disorders, which were subsequently confirmed by a psychiatrist (e.g., Allely et al., 2013; Marvick et al., 2013; Puckering et al., 2014). While this study elucidates important longitudinal connections, a limitation is that the clinical evaluation conducted by psychiatrists was limited to children who met the DAWBA cut‐off for mental disorders, potentially leaving unidentified cases, as also underscored by the researchers. The other group of longitudinal studies that included mental disorders as outcomes, are genetic high‐risk studies following younger siblings of probands with autism spectrum disorder (ASD). A review of these studies concluded that child characteristics during early parent‐child interactions, such as low attentiveness, negative affect, and less advanced vocalizations, predicted later ASD diagnosis, while relational characteristics of parents had limited predictive value (Wan et al., 2019). While these studies offer important insights into early relational signs of ASD, the outcome of interest is primarily ASD symptoms and diagnosis, hindering comparison across mental disorders. Also, despite the high heritability of ASD, many cases are sporadic without a recent family history (Yoon et al., 2021), making it unclear whether the findings from these sibling studies can be generalized to the broader ASD population (Szatmari et al., 2016).

Overall, various longitudinal studies indicate that dysfunctions in the early parent‐child relationship predict children's mental health problems at later ages. However, only a few of these studies focused on mental disorder diagnoses as outcomes. Consequently, it is still unclear whether and to what extent specific characteristics of the parent‐child relationship in the first years of childhood are predictive of more severe mental health problems that lead to clinical referral and meet the diagnostic criteria for mental disorders. The aim of the present investigation was therefore to examine such potential connections. This aim was pursued through a longitudinal child cohort study embedded within the Copenhagen Child Cohort (CCC2000; Skovgaard et al., 2008; Olsen et al., 2020). As part of this study, participants underwent comprehensive assessments of their parent‐child relationships at 18‐months of age and were subsequently monitored for mental disorder diagnoses across childhood and adolescence.

Based on the cited research, we hypothesized that dysfunctional characteristics of the early parent‐child relationship would be associated with a higher likelihood of being diagnosed with a mental disorder at later ages. However, due to the limited amount of prior research directly comparable to the present investigation, we refrained from making a priori predictions about specific diagnoses.

## METHODS

### Sample and design

This study was part of the CCC2000, a birth cohort study aimed at prospectively studying psychopathology from birth onwards (Olsen et al., 2020). At 18‐months of age a nested in cohort sub‐sample of 296 children participated in an in‐depth investigation of the parent‐child relationship and mental health (Skovgaard, Olsen, et al., 2005; Skovgaard et al., 2008). This sub‐cohort consisted of two sub‐samples. One subsample was a random sample (*n* = 210) from the CCC2000, recruited equally from geographical areas with high, medium, and low average incomes. The other subsample (*n* = 86) was recruited among children whose community health nurse had recorded concerns in their infancy health records. In Denmark, community health nurses conduct home visits to all families with newborns to assess the physical and mental development of the infant. This assessment involves evaluating whether there is a concern regarding the child's development or the family situation. The formation of the CCC2000 and the recruitment procedures are also described in prior publications (Olsen et al., 2020; Skovgaard, Olsen, et al., 2005; Skovgaard et al., 2007). To be included in the present investigation, the children were required to have competed the assessment at 18‐months of age and have a valid person identification number allowing linkage with outcome data from the Danish registers. To temporally distinguish the predictors from the outcomes, it was also required that the child had not been diagnosed with a mental disorder prior to the time of the 18‐month assessment, which is almost always the case anyhow this early in childhood (Koch et al., 2021). The CCC2000 is followed prospectively using unique personal identification numbers that enabled linkage with the national health registers in Denmark. For this study, the follow‐up period ended on December 31, 2016, at which time the participants were between the ages of 16–17. All the data was stored on a secure platform under Statistics Denmark, where the final data‐analyses were completed in December 2023. The study was approved by the Data Protection Agency in the Capital Region (CSU‐FCFS‐2016‐004, I‐Suite nr. 04544), as well as the Regional Ethics Committee of Copenhagen (protocol 16023242). Written informed consent was obtained from all parents who participated with their children when the children were 18‐months old. The study adhered to the rules of Statistics Denmark, which aim to prevent indirect identification by prohibiting the presentation of microdata, defined as cell sizes of three or below.

### Assessment of the early parent‐child relationship

At 18 months of age, the children and their parents were assessed using the Parent‐Child Early Relational Assessment (PC‐ERA; Clark, 1985, 1999). The PC‐ERA is an observational scoring system that assesses parent, child, and dyad characteristics of the early parent‐child relationship. The parent and child were video‐recorded during two scenarios: free‐play and mealtime. These recordings were then used to rate 65 items (29 parent items, 28 infant items, and 8 dyadic items) on a five‐point Likert scale where low scores (1–2) indicate an area of concern, middle score (3) some concern, and high scores (4–5) an area of strength. In accordance with the factors validated by Clark (1999), the items were organized into eight scales: (a) Parental Positive Affective Involvement and Verbalization; (b) Parental Negative Affect and Behavior; (c) Parental Intrusiveness, Insensitivity, and Inconsistency; (d) Infant Positive Affect, Social, and Communicative Competence; (e) Infant Quality of Play, Interest, and Attention Skills; (f) Infant Dysregulation and Irritability; (g) Dyadic Mutual Enjoyment and Reciprocity; and (h) Dyadic Tension and Disorganization. The PC‐ERA ratings were completed by child psychiatrists and developmental psychologists with expertise in infant and toddler mental health. The inter‐rater reliability of the PC‐ERA has been confirmed in a subsample (Skovgaard, Houmann, et al., 2005).

### Outcomes

The outcomes were mental disorders diagnosed during the follow‐up period. Mental disorders were analyzed both as a broad category, reflecting the presence or absence of mental disorders, as well as through specific diagnostic outcomes. Among mental disorders prevalent in children and adolescents (Polanczyk et al., 2015; Vasileva et al., 2021), ADHD, ASD, and emotional disorders were selected as outcomes, as these are the most common diagnostic groups among referred children and adolescents (Dalsgaard et al., 2020), ensuring palpable groups for data‐analysis.

Data on mental disorders during the follow‐up period were derived from the National Patient Registry and Central Psychiatric Registry. These registers contain information on all contacts in outpatient, inpatient, and emergency settings in hospitals in Denmark, including the date of contact and diagnoses rendered. In Denmark, healthcare is publicly funded through national income tax, thus access to health care is not biased by income and health insurance systems. For this reason, the Danish registers are widely used for research purposes (Schmidt et al., 2015). Mental disorders in children and adolescents are diagnosed in both inpatient and outpatient hospital settings and require thorough diagnostic assessment as conducted by a medical doctor following the diagnostic criteria of the International Classification of Diseases, with the 10th version being applicable to the time interval of the present investigation (ICD‐10; WHO, 1994). Studies have supported the validity of mental disorders recorded in Danish registers, including ADHD (Mohr‐Jensen et al., 2016), ASD (Lauritsen et al., 2010), obsessive‐compulsive disorder (Nissen et al., 2017), and major depression (Frederiksen et al., 2021). A nationwide study concluded that the overall rate of mental disorders recorded in Danish registers for children and adolescents was largely comparable to rates in studies using other modes of assessment, thus supporting the coverage of the Danish registers (Dalsgaard et al., 2020).

### Covariates

Child mental health problems at the time of the 18‐months investigation was assessed using the Child Behavior Checklist 1½‐5 (CBCL 1½‐5; Achenbach & Rescorla, 2000). This measure contains 100 items describing various mental health problems, which parents are asked to rate in terms of how well they describe their child within the last two months. The total problem score, representing the sum of all items, was used to determine the overall degree of mental health problems at 18‐months of age. Information on parental mental disorder in early childhood was obtained by combining two variables which were obtained as part of the CCCC2000 and the nested cohort study: (a) parental mental disorders within the last three years as recorded in The National Patient Registry and the Central Psychiatric Registry, as well as (b) responses to a self‐report item from the Mannheim Parent Interview (MPI; Esser et al., 1989) asking the parents to state whether they had a mental disorder requiring clinical treatment (Skovgaard et al., 2008).

### Statistical analyses

Cox proportional hazards regression analyses were used to examine each PC‐ERA scale as a predictor of mental disorders. Effect sizes were hazard ratios (HR) with 95% confidence intervals (CIs). A HR below 1 indicated that a lower PC‐ERA score (i.e., more dysfunction) was associated with the outcome, and vice versa for values above 1. Censoring was age at the end of the follow‐up period, death, or emigration, whichever came first, and time to event was age at mental disorder diagnosis. The outcomes were the presence of any mental disorder as well as specific mental disorders including emotional disorders, ADHD and ASD. To test the proportional hazard assumption, Schoenfeld residual tests were computed for each predictor variable, as well as the total cox model. Given the variation in sex ratios across specific mental disorders (Dalsgaard et al., 2020) as well as research pointing to different parenting styles based on the sex of the child (Mesman & Groeneveld, 2018), all cox models were adjusted for sex, defined as sex assigned at birth. To prevent redundant testing, associations between PC‐ERA dimensions and mental disorders were analyzed in two steps. Initially, each PC‐ERA scale was examined as potential predictor of the outcomes. Subsequently, the PC‐ERA scales that were identified as significant predictors were further analyzed in additional Cox‐models that included two potential confounders. Parental mental disorder and early mental health problems of the child were considered as potential confounders, as both have been found to impact the parent‐child dynamics in early childhood (Garner et al., 2013; Jacobson, 2016) as well as predict children's mental disorders (McLaughlin et al., 2012; Thapar & Rutter, 2015). All data analyses were performed using STATA‐18, and graphs were created using a Microsoft Excel radar chart template.

## RESULTS

In total, 295 children were included in this follow‐up study. Descriptive characteristics of the sample at baseline and follow‐up are provided in Table 1. There was no attrition due to death. However, 10 children (3%) emigrated with their families prior to the age of 10 but were retained in the analyses until the date of emigration. The remaining 285 children (97%) were retained until the end of the follow‐up, at which time they were between the ages of 16 and 17 (mean = 16.46 years, SD = 0.29). In total, the follow‐up for this nested child cohort contained 4832 person‐years. During the follow‐up period, 52 (17.6%) children were diagnosed with mental disorders. Descriptive characteristics on mental disorders diagnosed during the follow‐up period are provided in Table 1. Emotional disorders, including anxiety disorders, mood disorders, eating disorders, and attachment disorders (ICD‐10: F30‐39, F40‐49, F50, F93, F94.1‐2), represented the largest group of mental disorders, followed by ASD (ICD‐10: F84) and ADHD (ICD‐10: F90) (see Table 1). Only nine children were diagnosed with a mental disorder that did not belong to any of these three diagnostic groups. These other mental disorders were too few and too diverse to constitute a meaningful separate outcome category. However, the broad outcome, reflecting the presence of mental disorder, included all the mental disorders that were diagnosed during the follow‐up period (Table 1). Regarding comorbidity, six children with ASD were also diagnosed with emotional disorders. None of the children with ADHD were diagnosed with concurrent disorders within the main group of emotional disorders. The number of children diagnosed with both ASD and ADHD was below the minimum threshold of observations required for presenting microdata, in accordance with the rules of Statistics Denmark, which the study was obligated to adhere to.

**TABLE 1:** Demographics and parent‐child relationship characteristics at 18‐months of age, and mental disorders subsequently diagnosed in childhood and adolescence.

	Mean (*SD*)	*n* (%)
Baseline characteristics		
Child's sex assigned at birth (female)		139 (47.6%)
Child's age (months)	18.29 (0.56)	
Mother's age (years)	31.59 (4.97)	
Father's age (years)	33.81 (5.41)	
Parental mental disorder		52 (17.6%)
Child mental health problems (CBCL total problem score)	26.84 (0.70)	
Early parent‐child relational assessment (PC‐ERA)		
Parent dimensions:		
Parental positive affective involvement and verbalization	4.40 (0.57)	
Parental negative affect and behavior	4.84 (0.28)	
Parental intrusiveness, insensitivity, and inconsistency	4.56 (0.43)	
Infant dimensions:		
Infant positive affect	4.22 (0.68)	
Infant quality of play	4.52 (0.46)	
Infant dysregulation	4.65 (0.54)	
Dyadic dimensions:		
Dyadic mutual enjoyment and reciprocity	4.03 (0.79)	
Dyadic disorganization and tension	4.39 (0.58)	
Mental disorders diagnosed during the follow‐up (between 18‐months and 17 years of age)		
Mental disorder diagnosed during follow‐up^1^		52 (17.6%)
Mean age of at first mental disorder diagnosis	10.01 (4.34)	
Main diagnostic categories:		
Emotional disorders (F30‐39, F40‐49, F50, F93, F94.1‐2)		25 (8.5%)
Attention deficit hyperactivity disorders (F90)		15 (5.1%)
Autism spectrum disorders (F84)		15 (5.1%)

*Note*: Included all ICD‐10 mental disorders diagnosed during the follow‐up period: F00‐19, F30‐39, F40‐49, F50, F62, F70‐79, F80‐83, F84, F89.9, F90, F93, F94.1‐2, F95.

Abbreviations: ADHD, attention deficit hyperactivity disorder (F90); ASD, autism spectrum disorders (F84); CBCL, Child Behavior Checklist 1½‐5 (Achenbach & Rescorla, 2000); PC‐ERA, Parent Child Early Relational Assessment (Clark, 1985).

**FIGURE 1:**
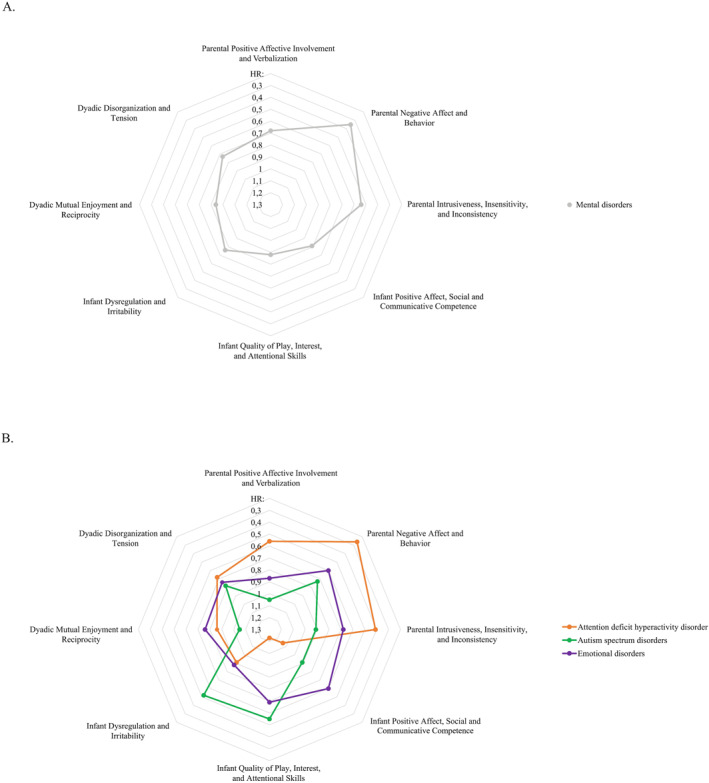
Parent, child, and dyadic characteristics of the early parent‐child relationship at 18‐months of age in children who were subsequently diagnosed with mental disorders in childhood and adolescence. (A) any mental disorders; (B) specific mental disorders. The further away from the inner circles the hazard ratio (HR) is, the more maladaptive the early relational dimension is.

In the first line of Cox models (Table 2, Figure 1A), each of the parent, child, and dyadic dimensions of the PC‐ERA were tested as predictors, while adjusting for sex assigned at birth. Two parent dimensions were significantly associated with a higher likelihood of the child being diagnosed with a mental disorder during the follow‐up period: Parent Negative Affect and Behaviors (HR = 0.35, 95% CI [0.17, 0.71], *p* = 0.004), and Parent Intrusiveness, Insensitivity, and Inconsistency (HR = 0.57, 95% CI [0.32, 0.99], *p* = 0.045). As indicated by the HRs, each lower point on these scales was, on average, associated with a 65% and 43% higher likelihood, respectively, of the child being diagnosed with a mental disorder at later ages.

**TABLE 2:** Parent, child and dyadic characteristics of the early parent‐child relationship as predictors of mental disorders subsequently diagnosed in childhood and adolescence.

PC‐ERA predictors	Mental disorder		Specific mental disorders					
			ADHD		ASD		Emotional disorders	
	HR (95% CI)	*p*	HR (95% CI)	*p*	HR (95% CI)	*p*	HR (95% CI)	*p*
Parental scales								
Parental positive affective involvement and verbalization	0.68 (0.45–1.04)	0.077	0.56 (0.27–1.17)	0.126	1.05 (0.42–2.58)	0.921	0.87 (0.45–1.66)	0.667
Parental negative affect and behavior	0.35 (0.17–0.69)	0.003**	0.26 (0.07–0.94)	0.039*	0.73 (0.15–3.43)	0.686	0.60 (0.19–1.86)	0.374
Parental intrusiveness, insensitivity, and inconsistency	0.54 (0.31–0.95)	0.033*	0.41 (0.14–1.13)	0.085	0.91 (0.28–2.89)	0.868	0.68 (0.29–1.58)	0.370
Infant scales								
Infant positive affect, social and communicative competence	0.81 (0.54–1.21)	0.297	1.14 (0.51–2.58)	0.746	0.91 (0.42–1.97)	0.819	0.60 (0.35–1.04)	0.070
Infant quality of play, interest, and attentional skills	0.88 (0.49–1.57)	0.669	1.23 (0.36–4.20)	0.743	0.55 (0.21–1.43)	0.221	0.69 (0.32–1.48)	0.340
Infant dysregulation and irritability	0.76 (0.51–1.14)	0.184	0.91 (0.38–2.18)	0.831	0.52 (0.30–0.91)	0.023*	0.88 (0.46–1.72)	0.717
Dyad scales								
Dyadic mutual enjoyment and reciprocity	0.84 (0.60–1.17)	0.299	0.86 (0.45–1.62)	0.635	1.05 (0.54–2.05)	0.892	0.76 (0.47–1.21)	0.243
Dyadic disorganization and tension	0.73 (0.47–1.14)	0.116	0.68 (0.30–1.51)	0.340	0.78 (0.34–1.82)	0.570	0.74 (0.40–1.40)	0.361

Abbreviations: ACBCL, Child Behavior Checklist 1½‐5 (Achenbach & Rescorla, 2000); ADHD, attention deficit hyperactivity disorder (F90); ASD, autism spectrum disorders (F84); PC‐ERA, Parent Child Early Relational Assessment (Clark, 1985).

* <0.05, ** <0.01.

Across the three diagnostic categories (Table 2. Figure 1B), only ADHD was predicted by relational characteristics of the parent, with Parental Negative Affect and Behaviors (HR = 0.26, 95% CI [0.14, 0.94], *p* = 0.039) emerging as a significant predictor. On average, each lower point on this scale conferred a 74% increased likelihood of the child being diagnosed with ADHD. In contrast, early relational characteristics pertaining to the child were only predictive of ASD. Child characteristics involving Dysregulation and Irritability (HR = 0.52, 95% CI [0.30, 0.91], *p* = 0.023) predicted a diagnosis of ASD with each lower point conferring, on average, a 48% increased likelihood of this outcome. None of the early parent‐child relational dimensions significantly predicted emotional disorders. The significant predictors identified in the first line of cox‐analyses were subsequently tested in a second line of cox analyses that further adjusted for parental mental disorder and child mental health problems at baseline. As shown in Table 3, the early relational dimensions that were initially identified as significant predictors of the outcomes remained significant in this second line of cox‐analyses. All Schoenfeld's residuals tests supported the proportional hazard assumption.

**TABLE 3:** Early relational predictors of mental disorders while adjusting for sex assigned at birth, early child mental health problems and parental mental disorders.

Outcomes:	Regression models with statistics for each predictor	HR (95% CI)	*p*
Mental disorders	**Model 1.** Parental negative affect and behavior (PC‐ERA)	0.31 (0.15–0.64)	0.001**
	Sex assigned at birth	0.78 (0.45–1.37)	0.339
	Early child mental health problems (CBCL)	1.03 (1.01–1.06)	0.001**
	Parental mental disorder	2.03 (1.11–3.73)	0.022*
	**Model 2.**		
	Parental intrusiveness, insensitivity, and inconsistency (PC‐ERA)	0.55 (0.31–0.95)	0.033*
	Sex assigned at birth	0.76 (0.43–1.32)	0.332
	Early child mental health problems (CBCL)	1.03 (1.01–1.05)	0.004**
	Parental mental disorder	2.21 (1.21–4.06)	0.010**
ADHD	**Model 3.**		
	Parental negative affect and behavior (PC‐ERA)	0.22 (0.06–0.84)	0.027*
	Sex assigned at birth	0.15 (0.03–0.69)	0.014*
	Early child mental health problems (CBCL)	1.05(1.01–1.09)	0.009**
	Parental mental disorder	1.42 (0.44–4.53)	0.554
ASD	**Model 4**		
	Infant dysregulation and irritability (PC‐ERA)	0.51 (0.28–0.92)	0.024*
	Sex assigned at birth	1.01 (0.36–2.81)	0.983
	Early child mental health problems (CBCL)	1.01 (0.97–1.06)	0.579
	Parental mental disorder	2.38 (0.81–7.00)	0.117

Abbreviations: ADHD, attention deficit hyperactivity disorder (F90); ASD, autism spectrum disorders (F84); CBCL, Child Behavior Checklist 1½‐5 (Achenbach & Rescorla, 2000); PC‐ERA, Parent Child Early Relational Assessment (Clark, 1985).

* <0.05, ** <0.01.

## DISCUSSION

To our knowledge, this is the first longitudinal cohort study to explore parental‐, child‐, and dyadic characteristics of the early parent‐child relationship at 18 months of age as potential predictors of different mental disorders diagnosed in childhood and adolescence. Across the different dimensions pertaining to the early parent‐child relationship, parent negative affect and behaviors, as well as parent intrusiveness, insensitivity, and inconsistency, were associated with a higher likelihood of the child subsequently being diagnosed with a mental disorder. This finding resonates with earlier longitudinal studies, which similarly suggest that infants exposed to dysfunctional parenting, such as lower parental sensitivity, are more likely to have mental health problems later in life (e.g., Jacobvitz et al., 2022; Kok et al., 2013) and score in the clinical range for probable mental disorders (McAndie et al., 2022). Contrary to these investigations, the outcomes in the present study were mental disorders clinically diagnosed in hospital settings, which also allowed for direct comparison across different groups of mental disorders. Different patterns of results were observed across the main diagnostic groups. Significant associations were identified for ASD and ADHD, while no significant associations emerged for emotional disorders.

Among the different dimensions of early parent‐child relationship, only parent characteristics were predictive of ADHD. Children whose parents displayed negative affect and behaviors when they were 18 months of age were more likely to be diagnosed with ADHD at later ages. This result resonates with longitudinal studies demonstrating that parental abuse and neglect are associated with a higher risk of ADHD symptoms and diagnosis (e.g., Claussen et al., 2022; González et al., 2019; Thompson & Tabone, 2010; Young et al., 2011), even when adjusting for gene‐environment correlations (Warrier et al., 2021). In contrast to these studies, parental characteristics were in the present study examined at an earlier age and in the context of both child and dyadic characteristics. According to the transactional model, dysfunctional parenting may in part be influenced by behaviors exhibited by the child (Sameroff & Mackenzie, 2003). Based on the present findings, there were no immediate indications to suggest that the association between dysfunctional parenting and ADHD could be explained by early child characteristics. For instance, parent negative affect and behavior remained a significant predictor of ADHD when the children's early behavior problems were adjusted for. Also, none of the dysfunctional child or dyadic characteristics simultaneously predicted ADHD. In fact, children who were diagnosed with ADHD were at 18‐months of age largely comparable to children without this diagnosis. Thus, there were no direct indications that the parental characteristics that predicted ADHD could be explained by specific child characteristics. Given the possible role of dysfunctional parenting in ADHD (e.g., Claussen et al., 2022; González et al., 2019; Warrier et al., 2021), it is paramount to identify such exposure as early as possible. According to the present study, parental characteristics associated with ADHD may be identifiable already at 18 months of age. The findings therefore bear upon early interventions that aim to mitigate the risk of ADHD. Systematic observation and assessment of potential dysfunctional parenting characteristics, such as parental negative affect and behavior, may take place during routine home visits in the child's first years of life. Depending on the severity of these issues, interventions may range from simple parenting advice to more targeted approaches aimed at improving parenting. A meta‐analytic review concluded that behavioral parent training has a positive impact upon the quality of the parent‐child relationship as well as reduces children's ADHD symptoms (Doffer et al., 2023). However, in the included studies parent training was administered to parents of children already diagnosed with ADHD, and therefore not at an early premorbid stage. Thus, it remains to be established whether interventions targeting parenting very early in childhood have a preventive effect on ADHD.

In contrast to ADHD, ASD was predicted by child rather than parental characteristics. Children who exhibited more dysregulation and irritability during interactions with their parents at 18‐months of age were subsequently more likely to be diagnosed with ASD. That these child characteristics predicted ASD in the absence of any parental predictors generally concur with genetic high‐risk studies following younger siblings of ASD probands (Wan et al., 2019). The present findings extend this research by suggesting that similar results can be identified in a broader child population not confined to individuals with familial risk of ASD. Interventions based on video‐feedback to promote positive parenting have shown promise in reducing the risk of ASD (Green et al., 2017; Whitehouse et al., 2021). However, these interventions have been found to have limited effects on the child's positive affect and attentiveness during interactions with parents (Green et al., 2017; Whitehouse et al., 2021): characteristics that conceptually overlap with dysregulation and irritability, which predicted ASD in the present investigation. Consequently, it is unclear to what extent such relational dysfunctions can be changed and whether this would impact the efficacy of early interventions and the risk of ASD.

Strengths and limitations of this study should be considered. Among the strengths are the long follow‐up period as well as the observer‐based assessment, which provides a comprehensive profile of the early parent‐child relationship. Another strength is the high retention of participants, enhancing the representativeness of the findings by mitigating common biases associated with loss to follow‐up in child cohort studies, such as the underrepresentation of disadvantaged families (Perez et al., 2007; Wolke et al., 2009). However, certain limitations should also be acknowledged. Firstly, the assessment of the parent‐child relationship was limited to early childhood. Although research indicates consistency in the parent‐child relationship from infancy to adolescence (Bornstein & Putnick, 2021), it is not a static phenomenon. Additional measurements of the relationship at later ages would have allowed for insights into potential changes and how these may affect the risk of mental disorders. Secondly, the uneven sample sizes, with much fewer individuals with diagnoses than without, strained the statistical power of the study, making it more difficult to identify significant associations. Thirdly, the fact that the study was confined to the first 17 years of life may inherently have resulted in an underrepresentation of mental disorders that typically emerge in late adolescence and beyond, such as major mood disorders (Solmi et al., 2022). This timeframe may also account for the lack of comorbid emotional disorders in ADHD, as these more frequently occur at later ages (e.g., Biederman et al., 2010; Huh et al., 2011). Fourthly, as the study design was not genetically sensitive, it could not be determined whether the identified associations were due to shared genetic variance, hence reflecting a gene‐environment correlation. While some twin and genome‐wide studies have addressed this question (e.g., Daníelsdóttir et al., 2024; Samek et al., 2015; Warrier et al., 2021), most of these studies measure caregiving environments retrospectively at later ages, making them subject to recall biases. Therefore, considering the current findings, a key goal for genetically sensitive studies is to determine whether very early dysfunctions in the parent‐child relationship constitute causal risk factors, once genetic confounding is accounted for. Lastly, it should be noted that within‐disorder heterogeneity is likely to exist. For instance, although the parent dimensions were not predictive of ASD, it cannot be excluded that some individuals with ASD have experienced dysfunctional parenting early in childhood.

In conclusion, the findings suggest that specific child and adolescent mental disorders are preceded by dysfunctions in the parent‐child relationship, observable as early as the second year of life. Based on observation of parent‐child interaction at 18‐months of age, the study found that negative affect and behavior exhibited by the parent specifically predicted a diagnosis of ADHD while dysregulation and irritability exhibited by the child specifically predicted a diagnosis of ASD. That the early relational predictors of ASD and ADHD were markedly different underscores the potential significance of disorder‐specific pathways to these neurodevelopmental disorders. Improving such relational dysfunctions may contribute to the goals of early interventions that aim to mitigate the risk of such mental disorders.

## AUTHOR CONTRIBUTIONS

**Kristine Kahr Nilsson:** Conceptualization; Formal analysis; Investigation; Project administration; Visualization; Writing ‐ original draft. **Lasse Grønnebæk:** Conceptualization; Formal analysis; Software; Visualization; Writing ‐ review & editing. **Ole Karkov Østergård:** Conceptualization; Writing ‐ original draft; Writing ‐ review & editing. **Susanne Landorph:** Conceptualization; Data curation; Methodology; Writing ‐ review & editing. **Tine Houmann:** Conceptualization; Methodology; Project administration; Writing ‐ review & editing. **Else Marie Olsen:** Conceptualization; Data curation; Funding acquisition; Investigation; Methodology; Supervision; Writing ‐ review & editing. **Anne Mette Skovgaard:** Conceptualization; Data curation; Funding acquisition; Investigation; Methodology; Project administration; Writing ‐ review & editing.

## CONFLICT OF INTEREST STATEMENT

The authors have declared that they have no competing or potential conflicts of interest.

## ETHICAL CONSIDERATIONS

The study was approved by the Data Protection Agency in the Capital Region (CSU‐FCFS‐2016‐004, I‐Suite nr. 04544), as well as the Regional Ethics Committee of Copenhagen (protocol 16023242). Written informed consent was obtained from all parents who participated with their children when the children were 18‐months old.

## Data Availability

The data gathered in the CCC2000 study are classified as sensitive personal data and cannot be made publicly available due to current data protection regulations. These data are stored and linked with information from the Danish national registries and analyzed via Statistics Denmark [https://www.dst.dk/en]. Researchers interested in accessing the data can apply in collaboration with CCC2000 researchers by reaching out to the CCC2000 steering committee. For further information, please visit our website: [https://www.regionh.dk/CCC2000/English/Sider/About‐CCC2000‐in‐short.aspx].
